# Interim biomarker results for trontinemab, a novel Brainshuttle™ antibody in development for the treatment of Alzheimer’s disease

**DOI:** 10.1002/alz70859_104288

**Published:** 2025-12-25

**Authors:** Gregory Klein, Gil D. Rabinovici, Henrik Zetterberg, Matteo Tonietto, Tobias Bittner, Daria Rukina, Fabien Alcaraz, Carsten Hofmann, Maddalena Marchesi, Jakub Wojtowicz, Ruth Croney, David Agnew, João A. Abrantes, Franziska Schaedeli Stark, Silke Ahlers, Paul Delmar, Hanno Svoboda, Iris Wiesel, Luka Kulic

**Affiliations:** ^1^ F. Hoffmann‐La Roche Ltd, Basel Switzerland; ^2^ University of California, San Francisco, San Francisco, CA USA; ^3^ Institute of Neuroscience and Physiology, The Sahlgrenska Academy at the University of Gothenburg, Mölndal Sweden; ^4^ Genentech, Inc., South San Francisco, CA USA; ^5^ Roche Products Ltd, Welwyn Garden City United Kingdom; ^6^ Excelya Germany GmbH, Mannheim Germany; ^7^ F. Hoffmann‐La Roche Ltd, Basel, Basel Switzerland

## Abstract

**Background:**

Trontinemab is a novel amyloid‐targeting Brainshuttle™ monoclonal antibody specifically engineered for efficient transferrin receptor 1‐mediated transport across the blood–brain barrier. It is currently under evaluation in the Phase Ib/IIa Brainshuttle™ AD study (NCT04639050) in participants with mild cognitive impairment due to Alzheimer’s disease (AD) or mild‐to‐moderate AD.

**Method:**

The Brainshuttle™ AD study is a randomized, double‐blind, placebo‐controlled, multiple ascending dose study designed to investigate the safety, tolerability, pharmacokinetics, and pharmacodynamics of trontinemab following intravenous (IV) infusion. The study uses a staggered parallel‐group design, with participants recruited to four initial dose cohorts: 0.2 mg/kg (Cohort 1), 0.6 mg/kg (Cohort 2), 1.8 mg/kg (Cohort 3), and 3.6 mg/kg (Cohort 4). A minimum of 10 study participants per dose cohort are randomized in a 4:1 ratio to receive either trontinemab or placebo IV every 4 weeks for a total of seven doses in Part 1 of the study. In a dose‐expansion study (Part 2), an additional 60 participants are enrolled in each of Cohorts 3 and 4. Biomarker results, including global and regional amyloid positron emission tomography (PET), volumetric magnetic resonance imaging (MRI), cerebrospinal fluid (CSF), and plasma will be presented. Plasma and CSF samples are analyzed using the Roche Elecsys NeuroToolKit.

**Result:**

An interim analysis (data snapshot: Sep 2, 2024) revealed dose‐dependent amyloid plaque lowering and downstream effects on biomarkers across all active doses. Part 1 Cohort 4 exhibited very rapid amyloid depletion: –89 centiloids after 12 weeks (n=13), and –107 centiloids after 28 weeks (n=12). CSF downstream markers including total tau, phosphorylated tau181, and neurogranin were reduced by 30%, 34%, and 29%, respectively by Week 25 in the 3.6 mg/kg dose cohort in Part 1. Updated results from the most recent data snapshot will include available 28‐week PET data as well as MRI, CSF, and plasma results from Part 2 of the study.

**Conclusion:**

Preliminary results from the ongoing Brainshuttle™ AD study suggest that rapid and deep amyloid reduction can be achieved in most participants at 1.8 and 3.6 mg/kg doses within ≤28 weeks. New biomarker data will be presented to further evaluate trontinemab’s continued development as a potential AD treatment.

